# Psychological factors associated with sensory processing sensitivity in caregiving contexts: A protocol for a systematic review and meta-analysis

**DOI:** 10.1371/journal.pone.0323721

**Published:** 2025-05-30

**Authors:** Tadgh Connery, Megan Doyle, Mike Murphy, Annalisa Setti

**Affiliations:** 1 School of Applied Psychology, University College Cork, Cork, Ireland; 2 Environmental Research Institute, University College Cork, Cork, Ireland; Xiamen University - Malaysia Campus: Xiamen University - Malaysia, MALAYSIA

## Abstract

**Background:**

Caregiving, in both professional and informal settings, is a stressful role linked to burnout, anxiety and other negative mental health outcomes. Both environmental factors, such as perceived excessive workload and responsibility, interpersonal conflict, and poor communication, as well as personality factors, such as extraversion, agreeableness and neuroticism, have been associated with negative psychological outcomes in carers. Sensory Processing Sensitivity (SPS) is a temperament trait associated with poor mental health, characterised by high empathy and reactivity to environmental stimuli. Highly Sensitive People (HSPs) score highly in SPS and account for approximately 30% of the world’s population. SPS has been shown to be a marker of differential susceptibility, which predisposes individuals to feeling the harmful effects of negative environments and to flourish in positive ones. Given the stressful nature of both professional and informal caring environments, it is of interest to examine the experience of HSPs, those who score highly in SPS, within caregiving roles. The research examining SPS’ psychological correlates in caregivers has yet to be synthesised. The current study aims to systematically review the psychological correlates of SPS in caregiving populations and, where possible, to test the strength of these associations through meta-analysis.

**Method:**

A systematic search of quantitative and mixed-methods studies, written in English and published since 1997, the year in which the HSP Scale was first validated, will be conducted across 11 databases: Web of Science, Scopus; EBSCO Academic Search Complete; APA PsycINFO; PubMed; MEDLINE; CINAHL Plus with Full Text; APA PsycArticles; Business Source Complete; SocINDEX with Full Text; and ERIC. Google Scholar will also be searched, and the first 200 records by relevance will be screened for eligibility. Forward and backward citation searches of included records will be also performed. MD will search for records, which will be screened for eligibility by MD and TC. Data extraction and quality assessment, using the Joanna Briggs Inventory Risk of Bias tool, will be performed by TC and MD, with doubts and discrepancies being resolved through discussion with AS and MM. Data will be synthesised narratively and, where sufficient data are available, correlational meta-analysis will be conducted using RStudio. The Preferred Reporting Items for Systematic Reviews and Meta-Analyses (PRISMA) guidelines were followed in the development of this protocol. The protocol has been registered in PROSPERO (CRD42024524463).

**Discussion:**

This review will present a systematic overview of psychological factors associated with SPS in caregivers. It will identify psychological outcomes associated with SPS, as well as risk factors and protective factors that may be targeted to reduce negative outcomes for caregivers. Identifying these factors will allow for a better understanding of the HSPs’ experience of caregiving and consequently aid in enhancing this experience.

## Introduction

Caregiving working environments are often characterised as being high-pressure, with high expectations, minimal empowerment and excessive responsibility contributing to high stress levels [1]. In a sample of 118 Polish nurses, 90.7% believed that stress was an integral part of the nursing profession [2]. Indeed, excessive occupational stress, stemming from burdensome workload, the stress of patient care, stressful work environment and stress related to management and interpersonal relationships, is present in nurses [3] and has been linked to poor wellbeing indicators in professional caregivers [4,5].

Meta-analytic evidence from 25 cross-sectional studies reveals prevalence rates of 32%, 40.6% and 32% for anxiety, stress and depression respectively, in nurses during the Covid-19 pandemic [6]. Across 19 studies, pooled prevalence rates of 28.1% for high emotional exhaustion, 25.4% for depersonalisation and 39.7% for low personal accomplishment were found in Chinese nurses [7]. The presence of poor mental health may contribute to the 11.3% attrition rate observed in nurses across 2019 in the USA [8].

But it is not just professional carers who report negative mental health outcomes. Home carers, people who provide care at home for relatives or loved ones who are sick, have disabilities or who cannot care for themselves, are also prone to experiencing poor mental health [9], something that has only been exacerbated in wake of the Covid-19 pandemic [10]. In a sample of 2,222 Australian adults who reported providing home care to others for more than five hours per week, caregivers were seen to experience significantly poorer mental and physical health, as well as greater financial stress, more responsibility, and less perceived social support than non-caregivers [11]. The importance of environmental factors is highlighted by this study’s mediation models, which found caregivers’ poorer mental and physical health to be explained by low levels of social support and high levels of conflict with family members. And so, while the professional care work poses environmental risks, so too, it seems, does informal, home care.

While environmental factors are linked to poor wellbeing indicators in carers, personality factors also play a role. Burnout has been positively associated with neuroticism [12] and negatively associated with professional quality of life [13]. A study of 682 Italian healthcare workers found dysfunctional coping to negatively correlate with extraversion, agreeableness, conscientiousness and openness, and positively associated with neuroticism [14]. For non-frontline workers, problem-focused coping positively correlated with extraversion, agreeableness and openness, while emotion-focused coping was positively associated with openness, and negatively associated with conscientiousness [14]. In frontline workers, problem-focused coping was positively linked with extraversion, conscientiousness, agreeableness and openness, with the latter two traits positively correlating with emotion-focused coping [14]. Thus, not only does personality seem to be associated with caregivers’ work-related mental health outcomes, but also in how they respond to occupational stress.

A psychological trait that has recently garnered attention within the literature is sensory processing sensitivity (SPS); a heritable temperament trait, present in approximately 30% of the world’s population, characterised by high degrees of empathy, emotional reactivity to, and cognitive processing of, sensory stimuli and environments [15]. Traditionally, it is conceptualised as a three-factor trait, comprising of: ease of excitation, which refers to sensitivity to overstimulation; low sensory threshold, which represents discomfort towards sensory stimuli; and aesthetic sensitivity, referring to sensitivity to subtleties and aesthetic qualities of one’s environment [16]. Low sensory threshold and ease of excitation tend to share associations with negative mental health outcomes, such as negative emotionality, anxiety and depression, while aesthetic sensitivity consistently correlates with positive affect and self-esteem [17]. SPS itself has been positively associated with depression [18], negative affect [19] and stress [20].

SPS has been proposed as a marker of differential susceptibility to both supportive and unfavourable environments, by which HSPs are disproportionately more responsive and reactive to negative, vulnerability-promoting environments, and to positive, growth-enhancing environments [21]. Recent research tested SPS as a marker of differential susceptibility in a population-based sample of 300 participants, and found while SPS was positively associated with burnout, anxiety, depression, stress, health complaints and nonprescription medication use, differences emerged between HSPs and non-HSPs when their environments were accounted for [22]. Significant differences were found between HSPs’ and non-HSPs’ health outcomes across positive and negative environments, using social support and reported life history as proxies for the type of environment [22]. HSPs in favourable environments, with more social support and fewer negative life events, such as family death, domestic abuse and loss of work, reported greater life satisfaction and fewer negative mental health outcomes than non-HSPs [22]. Conversely, HSPs in negative environments reported worse health outcomes than non-HSPs [22].

Thus, the interaction between SPS and environment seems key in determining health outcomes [21,22]. Given the stressful nature of both professional [2] and informal [10] caring environments, it is of interest to examine the experience of HSPs within these roles. Despite SPS’ global prevalence rate of approximately 30% [15], the research examining its psychological correlates in caregiving settings has yet to be synthesised.

### Aim of the review

The review’s primary aim is to narratively synthesise existing research examining associations between SPS and psychological variables in caregivers. Where possible, the strength of these associations will be established using correlational meta-analysis. Given that SPS leaves individuals vulnerable to negative physical and mental health outcomes in unfavourable environments, it is important to identify what outcomes are associated with it in caregiving settings, so as to be able to provide targeted care, interventions and supervision/mentorship to highly sensitive caregivers. This review will also identify modifiable risk and protective factors, which will inform these interventions.

The proposed review will answer the following review question

What psychological factors are associated with SPS in caregivers?

### Objectives

1. The primary objective is to create a narrative systematic overview of SPS, within caregiving samples, by critically synthesising the psychological factors associated with it.
2. The secondary objective, where sufficient data are available, is to assess the strength of these associations using meta-analysis.

## Methods and analysis

The Preferred Reporting Items for Systematic Reviews and Meta-Analyses Protocols (PRIMSA-P) guidelines were followed when developing this protocol [23,24]. The protocol was registered with the International Prospective Register of Systematic Reviews (PROSPERO; CRD42024524463).

### Types of studies

Only quantitative studies, including experimental, quasi-experimental, observational and mixed-method studies, written in the English language, will be included. Qualitative studies, policy briefs or reports, opinion papers, commentaries, editorials, news, theoretical papers, conference presentations, literature reviews, systematic reviews and meta-analyses will be excluded. Non-English records will also be excluded.

### Types of participants

The review will include studies that recruit adult (aged 18 years and above) caregiver participants. For this review, caregivers are defined as: nurses, all healthcare workers, palliative care workers, home carers, informal carers, hospice care workers, nursing home care workers, speech and language therapists, occupational therapists, care assistants and first responders.

#### Patient and public involvement.

Participants were not directly involved or recruited for this review, given that the research examines previously published data.

### Types of outcome measures

All psychological correlates, including but not limited to psychological outcomes, risk factors and protective factors, of SPS will be considered outcomes for this review. Psychological outcomes will be considered primary outcome measures, while psychological risk factors, protective factors and other psychological correlates will be considered secondary outcome measures for this review. Factors not associated with SPS, or factors associated with SPS in non-caregivers will be excluded.

### Measures of effect

Given the diversity of measurement scales, there may be some variety in scores and outcomes identified. Thus, the frequency and prevalence of characteristics, as well as means, p-values, effect sizes and standard deviations scores will be included.

### Search method

A systematic search of 11 academic databases (Web of Science, Scopus; EBSCO Academic Search Complete; APA PsycINFO; PubMed; MEDLINE; CINAHL Plus with Full Text; APA PsycArticles; Business Source Complete; SocINDEX with Full Text; and ERIC) will be conducted using the following search string: (“sensory processing sensitivity” OR “sensory-processing-sensitivity” OR “highly sensitive person” OR “high sensitivity” OR “environmental sensitivity” OR “hsp”) AND (“professional caring role” OR “nursing” OR “nurses” OR “healthcare” OR “healthcare provider” OR “hospital care” OR “home care” OR “hospice care” OR “palliative care” OR “care provision” OR “provision of care” OR “care assistant” OR “nursing home care” OR “first responders”). Titles and abstracts will be searched for the search string terms. The utility of using Google Scholar within systematic reviews to supplement searches of academic databases has been well-documented, and recommendations on using Google Scholar when searching for eligible records state that focusing on the first 200–300 records is optimal, as the majority of sources after the 200^th^ record, on average, are grey literature [25]. Following these recommendations, Google Scholar will also be searched using the search string, and the first 200 records will be retrieved and screened for eligibility. MD will conduct all searches.

The search strategy was developed by TC, MM and AS in consultation with the UCC Academic Librarian. The search will be limited to studies published in English, given the team’s limited resources and language constraints, and to those published since 1997, the year in which the Highly Sensitive Person Scale was first validated [15].

Forward and backward citation searches of all included records will be conducted using Citationchaser, a widely used tool that makes use of Lens.org to search across over 250 million academic records [26]. TC will conduct citation-chasing and subsequent screening independently, with doubts being resolved through mutual discussion and consensus with MD, MM and AS.

### Study selection

MD and TC will independently screen identified records, first by title and abstract and then by full text. It is anticipated that only a small number of records will remain after screening, so data extraction will be conducted by two reviewers. Data will be extracted using a data extraction sheet designed by TC for the purpose of the review. MD will conduct 80% of the screening and data extraction, and TC will conduct 20%. Conflicts, doubts and discrepancies around records’ inclusion eligibility will be resolved through discussion and consensus with MM and AS. In the event that an agreement cannot be reached through mutual discussion and consensus, AS, as senior author, will resolve disagreements between the research team. The screening process, including reasons for excluding records, will be documented using a PRISMA flow diagram.

### Data extraction process

The following details will be extracted from included papers:

- Title
- Year of publication
- Authors
- Region
- Sample description
- Outcomes (correlates of sensory processing sensitivity)
- Measurement tools used for sensory processing sensitivity and correlates
- Means, standard deviations, effect sizes p-values of SPS and its correlates

#### Missing data.

In cases where information is missing or incomplete, the authors will attempt to contact the study authors. Should they be unavailable, the data will be excluded from analysis, and this will be addressed in the review’s discussion section.

### Risk of bias assessment

The Joanna Briggs Institute Critical Appraisal Tools will be used to assess the quality of evidence and methodological quality of included studies. The appropriate risk of bias tool (i.e., Checklist for Analytical Cross-Sectional Studies, Checklist for Quasi-Experimental Studies, Checklist for Randomised Control Trials) will be applied to assess each included record’s quality based on its study design. Quality assessment will be conducted for each record independently by MD and TC. Papers will be classified as being low, medium and high risk, depending on their rating for each criterion. TC’s and MD’s assessments will be compared, and an inter-rater reliability coefficient will be calculated using the kappa coefficient. Doubts and discrepancies between the team will be resolved through mutual deliberation and consensus between the research team.

If data or details pertaining to a study’s procedure are missing from the report, the relevant corresponding author/co-author will be contacted in an attempt to retrieve them. Failing this, the data will be excluded from analysis, and this will be addressed in the review’s discussion section.

### Data synthesis

Adhering to Cochrane guidelines [27], TC will conduct a narrative synthesis of the included studies and identify the prevalence of each psychological correlate across them. Sample characteristics will be described and measurement tools, for both SPS and its psychological correlates, will also be noted.

### Meta-analysis

Where at least four studies present associations between SPS and the same psychological correlate, each measured with the same respective scale, meta-analysis will be performed using the “metafor” and “robumeta” packages for RStudio. Effect sizes (pooled associations between SPS and psychological characteristics) and corresponding 95% confidence intervals will be calculated respectively. Statistical heterogeneity between the studies will be calculated using I^2^ values. An I^2^ statistic <25% will indicate low heterogeneity, an I^2^ statistic between 25–50% will indicate moderate heterogeneity and an I^2^ statistic >50% will represent high heterogeneity. A random-effects model will be used for highly heterogeneous studies (>50%) and, where the level of heterogeneity is not significant, a fixed-effects model will be applied to perform data pooling.

#### Subgroup analysis.

While it is anticipated that the total number of studies included will be low, subgroup analysis will be performed, stratifying groups by caregiver status (professional caregiver and non-professional caregiver) and caregiver occupation (i.e., Nurses, Doctors, Hospice caregivers, Informal caregivers) should sufficient data be available.

#### Assessment of publication bias.

Publication bias across included studies will be assessed using the visual examination of funnel plot and Egger’s test, should ten or more studies be eligible for inclusion. Asymmetry in the funnel plot may suggest possible publication bias, or other effects such as small-study effects. Should small study effects be the cause of this asymmetry, sensitivity analysis will be conducted to assess whether this effects the overall meta-analysis results.

#### Sensitivity analysis.

Sensitivity analysis will assess the robustness of pooled results, in terms of study characteristics and methodological quality, by removing some of the small-sample or low-quality studies. Should heterogeneity exist, sensitivity analysis will be re-run to remove studies with poor quality data or outliers.

## Discussion

This study aims to provide a comprehensive overview of factors associated with SPS in formal and informal caregivers, based on available quantitative evidence.

### Strengths and limitations of the study protocol

Strengths of systematic review protocol includes its adherence to PRISMA guidelines, its clear set of inclusion and exclusion criteria, and comprehensive search strategy, which includes a variety of academic databases and a search string developed by the research team in collaboration with a specialist academic librarian. The use of the Joanna Briggs Inventory risk of bias tools, which are well-established quality assessment tools, is also a strength as it allows for quality assessment to be tailored to each article’s study design. Given the research team’s limited resources, the current review’s exclusion of non-English records presents a limitation. While the number of relevant non-English records is anticipated to be low, this limitation introduces the possibility of publication and language biases.

### Implications of the study protocol

Given the breadth of research linking SPS to poor mental and physical health outcomes, as well as recent research which shows it to be a predisposing factor to negative outcomes in unfavourable environments, it is worth investigating its correlates within caregiving working environments, which are often perceived as being high-pressure working environments. This review will not only systematically capture the outcomes of SPS within caregiving populations, but it will also allow for an identification of the modifiable risk and protective factors that can be targeted to reduce negative outcomes for caregivers high in SPS.

While SPS remains a relatively new topic of research, there exists an impressive body of research that has conceptualised it as a trait. At present, the literature examining SPS in caregivers has yet to be systematically reviewed. The current review will further contribute to existing literature, and will lend itself to future research by identifying factors that are most strongly associated with SPS, ensuring that meaningful confounders are controlled for in future studies. Identifying modifiable factors to reduce negative outcomes of SPS, it will also aid in the development of relevant interventions. Finally, through dissemination to relevant stakeholders, it will raise awareness of SPS within caregivers and raise awareness of how to best support HSP care workers, formal and informal, to maintain positive wellbeing in the workplace and to continue providing effective care.

## Supporting information

S1 File Prismap_checklist_completed_tc. (DOCX)
